# The complete maternal mitochondrial genome sequences of two imperiled North American freshwater mussels: *Alasmidonta heterodon* and *Alasmidonta varicosa* (Bivalvia: Unionoida: Unionidae)

**DOI:** 10.1080/23802359.2018.1501307

**Published:** 2018-10-25

**Authors:** Aaron William Aunins, Cheryl L. Morrison, Heather S. Galbraith, Michael S. Eackles, William B. Schill, Timothy L. King

**Affiliations:** a Natural Systems Analysts, Under Contract to U.S. Geological Survey, U.S. Geological Survey Leetown Science Center, Kearneysville, WV, USA;; b U.S. Geological Survey Leetown Science Center, Kearneysville, WV, USA;; c Northern Appalachian Research Laboratory, U.S. Geological Survey Leetown Science Center, Wellsboro, PA, USA

**Keywords:** Anodontinae, *Alasmidonta heterodon*, dwarf wedgemussel, *Alasmidonta varicosa*, brook floater

## Abstract

The freshwater mussels *Alasmidonta heterodon* and *A. varicosa* historically inhabited rivers along the North American Atlantic coast from the Carolinas, U.S.A., to New Brunswick, CA. However, many populations have been extirpated, and *A. heterodon* is now federally listed in the U.S.A. as endangered, and both *A. heterodon* and *A. varicosa* are listed as vulnerable on the IUCN Red List. To facilitate genetic study of these species, we sequenced the complete female mitochondrial genomes of *A. heterodon* (15,909 bp; GenBank accession no. MG905826), and *A. varicosa* (15,693 bp; GenBank accession no. MG938673). Both mitogenomes contained 14 protein coding genes, 2 rRNA genes, and 22 tRNAs with the same gene order as reported for other members of the subfamily Anodontinae. When these two genomes were put into a phylogenetic context with other members of the Unionidae, they clustered together with other species in the subfamily Anodontinae, Tribe Anodontini.

Freshwater mussels are considered to be one of the most critically imperiled groups of organisms worldwide, due to a variety of anthropogenic stressors, with populations of even common species declining at dramatic rates (Lydeard et al. 2004). In light of these declines, critical research questions for freshwater mussel conservation and restoration include resolving mussel taxonomy and determining relatedness of individuals among populations (i.e., what are evolutionary significant units), identifying the effects of mussel declines on long-term population viability, and predicting response of mussels to further changes in the environment (National Native Mussel Conservation Committee 2016). However, genomic data are necessary to address these issues. Unfortunately, genomic resources are lacking for many freshwater mussel species (Saavedra and Bachère 2006). Here, we present the complete mitogenomes of *A. heterodon* and *A. varicosa* to facilitate genomic investigations of these species.

Genomic DNA of a single *A. heterodon* was extracted from mantle tissue of a specimen collected from the Delaware River, PA, in 2002. For *A. varicosa*, equal volumes of extracted DNA from four individuals collected in 2009 from a tributary of the Connecticut River, was pooled into one composite sample for library preparation. Extracted DNA from these specimens is stored at the Leetown Science Center (Kearneysville, WV). Extracted DNA for *A. heterodon* was prepared for paired-end sequencing (2 × 150 bp) using the NEBNext Ultra^TM^ DNA Library Prep Kit for Illumina^®^, dual indexed, and sequenced on a high output 300 cycle cartridge with other unrelated libraries on an Illumina NextSeq500^®^ (Illumina, San Diego, CA). The composite *A. varicosa* DNA sample was prepared for 200 bp single-end sequencing on the Ion Torrent Personal Genome Machine (PGM^TM^, Life Technologies, Foster City, CA), using the IonXpress^TM^ Plus gDNA Fragment Library kit, and sequenced on two separate runs utilizing 316 chips. All sequencing was performed at the Leetown Science Center (Kearneysville, WV).

Raw sequence data from all runs were imported into Qiagen CLC Genomics Workbench ver. 9 for analysis. The two *A. varicosa* PGM runs were pooled and analyze as a single set of sequences. All sequences were quality trimmed in CLC using a trimming parameter of 0.05. Separate *de novo* assemblies of 19,528,388 non-overlapping paired end reads for *A. heterodon*, and 4,681,811 single end reads of *A. varicosa* each yielded a contig corresponding to their respective mitogenomes. Circularity was confirmed by joining the ends of the contigs and looking for contiguous mapping of sequence reads across the contig ends. Annotation of the mitogenomes was initially performed using MITOS2 (Bernt et al. 2013) to identify tRNAs and approximate locations of protein coding genes. Next, DOGMA (Wyman et al. 2004) was used to identify start and stop codons of protein coding genes in conjunction with manual comparisons of putative *A. heterodon* and *A. varicosa* protein coding genes with other annotated unionid mitochondrial DNA sequences in GenBank.

The *A. heterodon* (15,909 bp; GenBank accession no. MG905826) and *A. varicosa* (15,693 bp; GB accession no. MG938673) mitogenomes each contain 14 protein-coding genes (one of which is the female mitochondria specific open reading frame FORF putatively involved in sex-determination, Guerra et al. 2017), 22 tRNAs, and large and small subunit ribosomal RNAs. Gene order was identical between the two species, with the difference in size accounted for primarily due to a larger putative control region (Breton et al. 2009) between *nad*5 and tRNA-Q in *A. heterodon*. To investigate the phylogenetic relationship of *A. varicosa* and *A. heterodon* relative to other unionids, we constructed a maximum likelihood tree based on an alignment of 13 protein coding genes (excluding FORF and HORF genes) from *A. varicosa* and *A. heterodon* plus 41 additional complete unionid mitochondrial genomes that were closest matches in GenBank, including representatives from five of the six subfamilies of Unionidae (Lopes-Lima et al. 2017), plus Margaritiferidae. Amino acid alignments were generated using MUSCLE (Edgar 2004) implemented in TranslatorX (Abascal et al. 2010). The aligned genes were concatenated and analyzed using PartitionFinder 2 to find the best-fit partitioning scheme and evolutionary model. The maximum likelihood analysis and branch support were assessed using 1000 ultrafast bootstrap resamplings (Hoang et al. 2018) in IQ-Tree (Nguyen et al. 2015). The families Margaritiferidae and Unionidae, as well as subfamilies Ambleminae and Rectidentinae were recovered (Figure 1). As expected, *A. varicosa* and *A. heterodon* grouped together in a clade containing taxa from the subfamily Anodontinae tribes Anodontini, and Cristarini, while tribe Lanceolarini representatives from far-East Asia formed a sister group clustering with *Acuticosta chinensis* (subfamily Unioninae: *insertae sedis*; Lopes-Lima et al. 2017). Generally, the two major clades reported by Lopes-Lima et al. (2017) were recovered (Anodoninae + Unioninae and Rectidentinae + Gonideinae + Ambleminae). However, taxa belonging to Gonideinae, tribe Lamprotulini, were found in three divergent clades, and other Unioninae *insertae sedis* taxa, also from far-East Asia and Russia were scattered throughout the tree.

**Figure 1.**
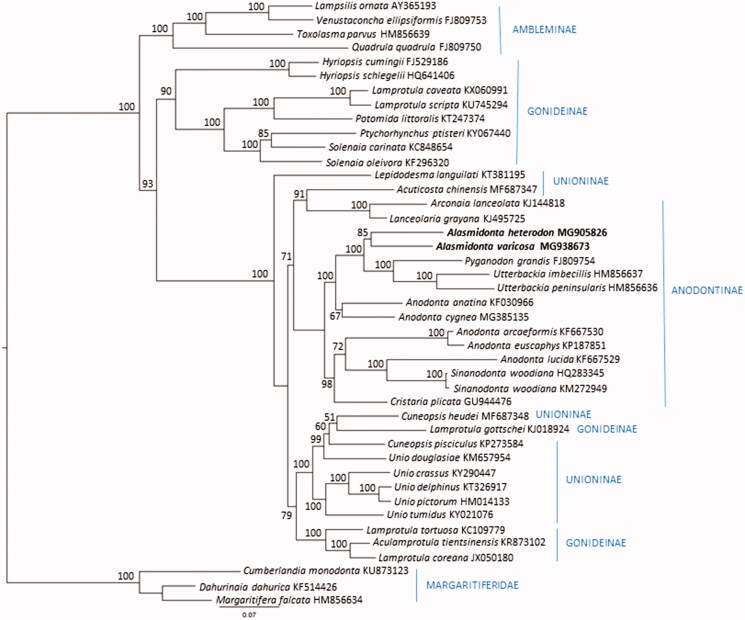
Partitioned maximum likelihood unrooted phylogenetic tree from a concatenated alignment of 13 protein coding genes (3655 amino acid positions, excluding FORF and HORF) for 43 unionid species. The maximum likelihood analysis was performed in IQ-Tree (Nguyen et al. 2015) utilizing the best corrected AIC score partitioning scheme (PartitionFinder2; Lanfear et al. 2017). Bootstrap support was assessed with 1000 ultrafast bootstrap resamplings in IQ-Tree (Hoang et al. 2018). GenBank accession numbers are included next to the species names, and subfamily and tribe designations follow (Lopes-Lima et al. 2017).
